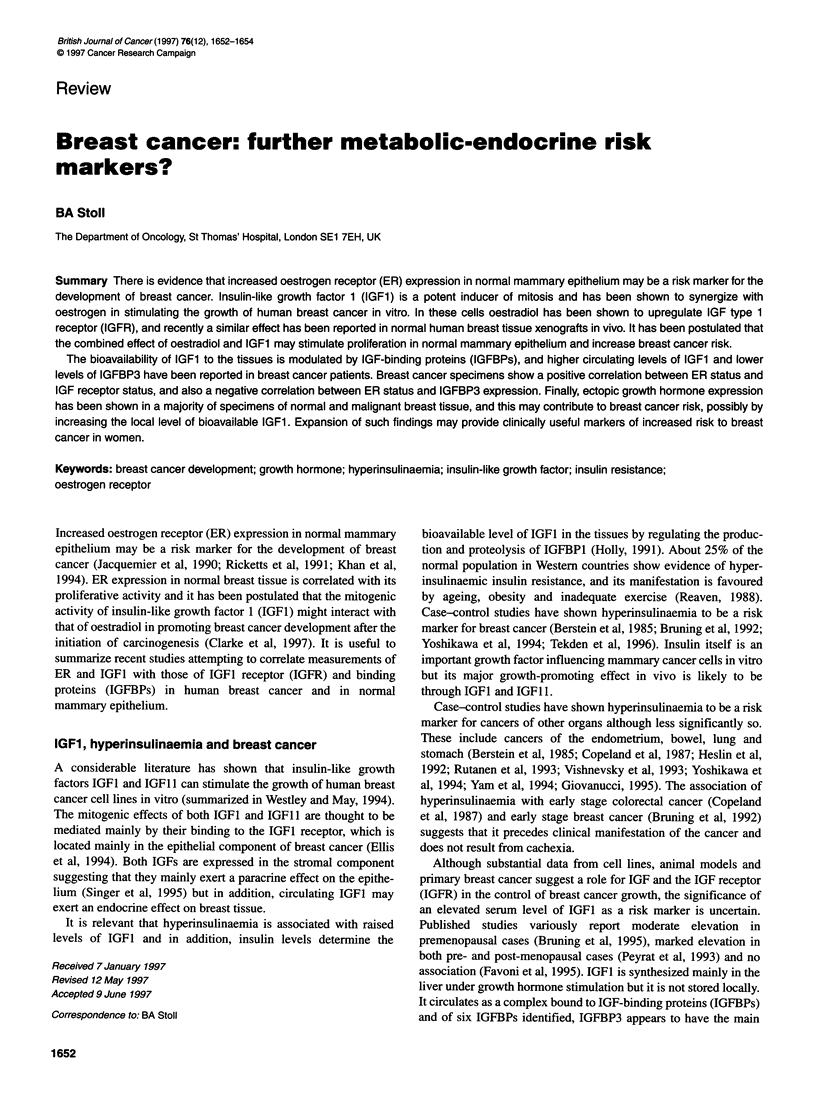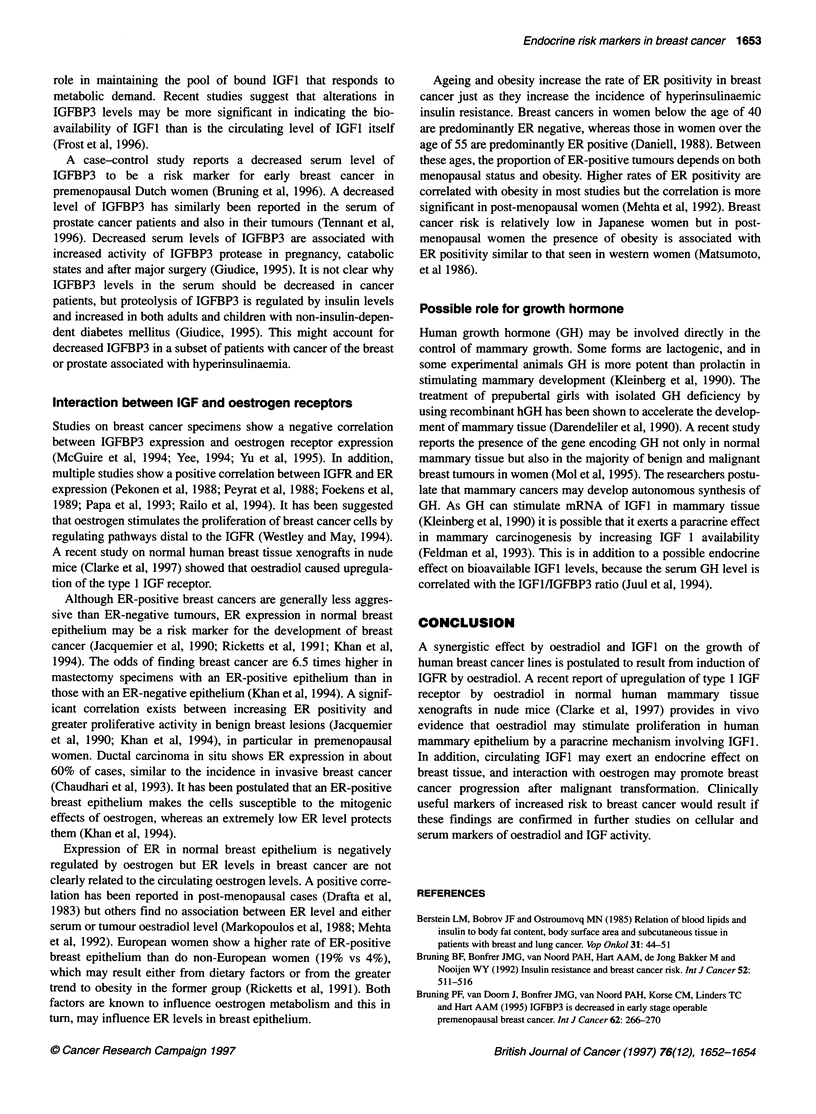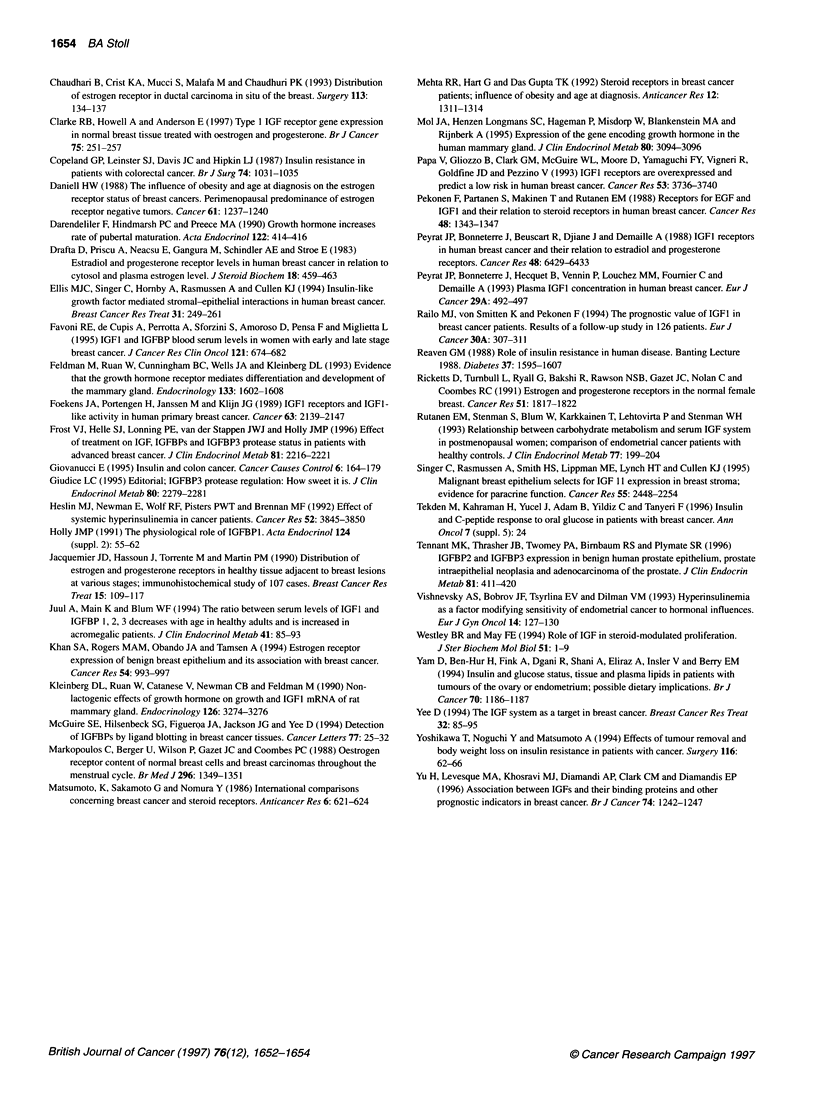# Breast cancer: further metabolic-endocrine risk markers?

**DOI:** 10.1038/bjc.1997.612

**Published:** 1997

**Authors:** B. A. Stoll

**Affiliations:** The Department of Oncology, St Thomas' Hospital, London, UK.

## Abstract

There is evidence that increased oestrogen receptor (ER) expression in normal mammary epithelium may be a risk marker for the development of breast cancer. Insulin-like growth factor 1 (IGF1) is a potent inducer of mitosis and has been shown to synergize with oestrogen in stimulating the growth of human breast cancer in vitro. In these cells oestradiol has been shown to upregulate IGF type 1 receptor (IGFR), and recently a similar effect has been reported in normal human breast tissue xenografts in vivo. It has been postulated that the combined effect of oestradiol and IGF1 may stimulate proliferation in normal mammary epithelium and increase breast cancer risk. The bioavailability of IGF1 to the tissues is modulated by IGF-binding proteins (IGFBPs), and higher circulating levels of IGF1 and lower levels of IGFBP3 have been reported in breast cancer patients. Breast cancer specimens show a positive correlation between ER status and IGF receptor status, and also a negative correlation between ER status and IGFBP3 expression. Finally, ectopic growth hormone expression has been shown in a majority of specimens of normal and malignant breast tissue, and this may contribute to breast cancer risk, possibly by increasing the local level of bioavailable IGF1. Expansion of such findings may provide clinically useful markers of increased risk to breast cancer in women.


British Journal of Cancer (1997) 76(12), 1652–1654
© 1997 Cancer Research Campaign

Review

### Breast cancer: further metabolic-endocrine risk markers?

**BA Stoll**

The Department of Oncology, St Thomas' Hospital, London SE1 7EH, UK

**Summary** There is evidence that increased oestrogen receptor (ER) expression in normal mammary epithelium may be a risk marker for the development of breast cancer. Insulin-like growth factor 1 (IGF1) is a potent inducer of mitosis and has been shown to synergize with oestrogen in stimulating the growth of human breast cancer in vitro. In these cells oestradiol has been shown to upregulate IGF type 1 receptor (IGFR), and recently a similar effect has been reported in normal human breast tissue xenografts in vivo. It has been postulated that the combined effect of oestradiol and IGF1 may stimulate proliferation in normal mammary epithelium and increase breast cancer risk.

The bioavailability of IGF1 to the tissues is modulated by IGF-binding proteins (IGFBPs), and higher circulating levels of IGF1 and lower levels of IGFBP3 have been reported in breast cancer patients. Breast cancer specimens show a positive correlation between ER status and IGF receptor status, and also a negative correlation between ER status and IGFBP3 expression. Finally, ectopic growth hormone expression has been shown in a majority of specimens of normal and malignant breast tissue, and this may contribute to breast cancer risk, possibly by increasing the local level of bioavailable IGF1. Expansion of such findings may provide clinically useful markers of increased risk to breast cancer in women.

**Keywords:** breast cancer development; growth hormone; hyperinsulinaemia; insulin-like growth factor; insulin resistance; oestrogen receptor

Increased oestrogen receptor (ER) expression in normal mammary epithelium may be a risk marker for the development of breast cancer (Jacquemier et al, 1990; Ricketts et al, 1991; Khan et al, 1994). ER expression in normal breast tissue is correlated with its proliferative activity and it has been postulated that the mitogenic activity of insulin-like growth factor 1 (IGF1) might interact with that of oestradiol in promoting breast cancer development after the initiation of carcinogenesis (Clarke et al, 1997). It is useful to summarize recent studies attempting to correlate measurements of ER and IGF1 with those of IGF1 receptor (IGFR) and binding proteins (IGFBPs) in human breast cancer and in normal mammary epithelium.

### IGF1, hyperinsulinaemia and breast cancer

A considerable literature has shown that insulin-like growth factors IGF1 and IGF11 can stimulate the growth of human breast cancer cell lines in vitro (summarized in Westley and May, 1994). The mitogenic effects of both IGF1 and IGF11 are thought to be mediated mainly by their binding to the IGF1 receptor, which is located mainly in the epithelial component of breast cancer (Ellis et al, 1994). Both IGFs are expressed in the stromal component suggesting that they mainly exert a paracrine effect on the epithelium (Singer et al, 1995) but in addition, circulating IGF1 may exert an endocrine effect on breast tissue.

It is relevant that hyperinsulinaemia is associated with raised levels of IGF1 and in addition, insulin levels determine the bioavailable level of IGF1 in the tissues by regulating the production and proteolysis of IGFBP1 (Holly, 1991). About 25% of the normal population in Western countries show evidence of hyperinsulinaemic insulin resistance, and its manifestation is favoured by ageing, obesity and inadequate exercise (Reaven, 1988). Case–control studies have shown hyperinsulinaemia to be a risk marker for breast cancer (Berstein et al, 1985; Bruning et al, 1992; Yoshikawa et al, 1994; Tekden et al, 1996). Insulin itself is an important growth factor influencing mammary cancer cells in vitro but its major growth-promoting effect in vivo is likely to be through IGF1 and IGF11.

Case–control studies have shown hyperinsulinaemia to be a risk marker for cancers of other organs although less significantly so. These include cancers of the endometrium, bowel, lung and stomach (Berstein et al, 1985; Copeland et al, 1987; Heslin et al, 1992; Rutanen et al, 1993; Vishnevsky et al, 1993; Yoshikawa et al, 1994; Yam et al, 1994; Giovanucci, 1995). The association of hyperinsulinaemia with early stage colorectal cancer (Copeland et al, 1987) and early stage breast cancer (Bruning et al, 1992) suggests that it precedes clinical manifestation of the cancer and does not result from cachexia.

Although substantial data from cell lines, animal models and primary breast cancer suggest a role for IGF and the IGF receptor (IGFR) in the control of breast cancer growth, the significance of an elevated serum level of IGF1 as a risk marker is uncertain. Published studies variously report moderate elevation in premenopausal cases (Bruning et al, 1995), marked elevation in both pre- and post-menopausal cases (Peyrat et al, 1993) and no association (Favoni et al, 1995). IGF1 is synthesized mainly in the liver under growth hormone stimulation but it is not stored locally. It circulates as a complex bound to IGF-binding proteins (IGFBPs) and of six IGFBPs identified, IGFBP3 appears to have the main

*Received 7 January 1997*
*Revised 12 May 1997*
*Accepted 9 June 1997*

*Correspondence to:* BA Stoll role in maintaining the pool of bound IGF1 that responds to metabolic demand. Recent studies suggest that alterations in IGFBP3 levels may be more significant in indicating the bioavailability of IGF1 than is the circulating level of IGF1 itself (Frost et al, 1996).

A case–control study reports a decreased serum level of IGFBP3 to be a risk marker for early breast cancer in premenopausal Dutch women (Bruning et al, 1996). A decreased level of IGFBP3 has similarly been reported in the serum of prostate cancer patients and also in their tumours (Tennant et al, 1996). Decreased serum levels of IGFBP3 are associated with increased activity of IGFBP3 protease in pregnancy, catabolic states and after major surgery (Giudice, 1995). It is not clear why IGFBP3 levels in the serum should be decreased in cancer patients, but proteolysis of IGFBP3 is regulated by insulin levels and increased in both adults and children with non-insulin-dependent diabetes mellitus (Giudice, 1995). This might account for decreased IGFBP3 in a subset of patients with cancer of the breast or prostate associated with hyperinsulinaemia.

### Interaction between IGF and oestrogen receptors

Studies on breast cancer specimens show a negative correlation between IGFBP3 expression and oestrogen receptor expression (McGuire et al, 1994; Yee, 1994; Yu et al, 1995). In addition, multiple studies show a positive correlation between IGFR and ER expression (Pekonen et al, 1988; Peyrat et al, 1988; Foekens et al, 1989; Papa et al, 1993; Railo et al, 1994). It has been suggested that oestrogen stimulates the proliferation of breast cancer cells by regulating pathways distal to the IGFR (Westley and May, 1994). A recent study on normal human breast tissue xenografts in nude mice (Clarke et al, 1997) showed that oestradiol caused upregulation of the type 1 IGF receptor.

Although ER-positive breast cancers are generally less aggressive than ER-negative tumours, ER expression in normal breast epithelium may be a risk marker for the development of breast cancer (Jacquemier et al, 1990; Ricketts et al, 1991; Khan et al, 1994). The odds of finding breast cancer are 6.5 times higher in mastectomy specimens with an ER-positive epithelium than in those with an ER-negative epithelium (Khan et al, 1994). A significant correlation exists between increasing ER positivity and greater proliferative activity in benign breast lesions (Jacquemier et al, 1990; Khan et al, 1994), in particular in premenopausal women. Ductal carcinoma in situ shows ER expression in about 60% of cases, similar to the incidence in invasive breast cancer (Chaudhari et al, 1993). It has been postulated that an ER-positive breast epithelium makes the cells susceptible to the mitogenic effects of oestrogen, whereas an extremely low ER level protects them (Khan et al, 1994).

Expression of ER in normal breast epithelium is negatively regulated by oestrogen but ER levels in breast cancer are not clearly related to the circulating oestrogen levels. A positive correlation has been reported in post-menopausal cases (Drafta et al, 1983) but others find no association between ER level and either serum or tumour oestradiol level (Markopoulos et al, 1988; Mehta et al, 1992). European women show a higher rate of ER-positive breast epithelium than do non-European women (19% vs 4%), which may result either from dietary factors or from the greater trend to obesity in the former group (Ricketts et al, 1991). Both factors are known to influence oestrogen metabolism and this in turn, may influence ER levels in breast epithelium.

Ageing and obesity increase the rate of ER positivity in breast cancer just as they increase the incidence of hyperinsulinaemic insulin resistance. Breast cancers in women below the age of 40 are predominantly ER negative, whereas those in women over the age of 55 are predominantly ER positive (Daniell, 1988). Between these ages, the proportion of ER-positive tumours depends on both menopausal status and obesity. Higher rates of ER positivity are correlated with obesity in most studies but the correlation is more significant in post-menopausal women (Mehta et al, 1992). Breast cancer risk is relatively low in Japanese women but in post-menopausal women the presence of obesity is associated with ER positivity similar to that seen in western women (Matsumoto, et al 1986).

### Possible role for growth hormone

Human growth hormone (GH) may be involved directly in the control of mammary growth. Some forms are lactogenic, and in some experimental animals GH is more potent than prolactin in stimulating mammary development (Kleinberg et al, 1990). The treatment of prepubertal girls with isolated GH deficiency by using recombinant hGH has been shown to accelerate the development of mammary tissue (Darendeliler et al, 1990). A recent study reports the presence of the gene encoding GH not only in normal mammary tissue but also in the majority of benign and malignant breast tumours in women (Mol et al, 1995). The researchers postulate that mammary cancers may develop autonomous synthesis of GH. As GH can stimulate mRNA of IGF1 in mammary tissue (Kleinberg et al, 1990) it is possible that it exerts a paracrine effect in mammary carcinogenesis by increasing IGF 1 availability (Feldman et al, 1993). This is in addition to a possible endocrine effect on bioavailable IGF1 levels, because the serum GH level is correlated with the IGF1/IGFBP3 ratio (Juul et al, 1994).

### CONCLUSION

A synergistic effect by oestradiol and IGF1 on the growth of human breast cancer lines is postulated to result from induction of IGFR by oestradiol. A recent report of upregulation of type 1 IGF receptor by oestradiol in normal human mammary tissue xenografts in nude mice (Clarke et al, 1997) provides in vivo evidence that oestradiol may stimulate proliferation in human mammary epithelium by a paracrine mechanism involving IGF1. In addition, circulating IGF1 may exert an endocrine effect on breast tissue, and interaction with oestrogen may promote breast cancer progression after malignant transformation. Clinically useful markers of increased risk to breast cancer would result if these findings are confirmed in further studies on cellular and serum markers of oestradiol and IGF activity.

### REFERENCES

Berstein LM, Bobrov JF and Ostroumovq MN (1985) Relation of blood lipids and insulin to body fat content, body surface area and subcutaneous tissue in patients with breast and lung cancer. *Vop Onkol* **31**: 44–51

Bruning BF, Bonfrer JMG, van Noord PAH, Hart AAM, de Jong Bakker M and Nooijen WY (1992) Insulin resistance and breast cancer risk. *Int J Cancer* **52**: 511–516

Bruning PF, van Doorn J, Bonfrer JMG, van Noord PAH, Korse CM, Linders TC and Hart AAM (1995) IGFBP3 is decreased in early stage operable premenopausal breast cancer. *Int J Cancer* **62**: 266–270

© Cancer Research Campaign 1997 

Chaudhari B, Crist KA, Mucci S, Malafa M and Chaudhuri PK (1993) Distribution of estrogen receptor in ductal carcinoma in situ of the breast. *Surgery* **113**: 134–137

Clarke RB, Howell A and Anderson E (1997) Type 1 IGF receptor gene expression in normal breast tissue treated with oestrogen and progesterone. *Br J Cancer* **75**: 251–257

Copeland GP, Leinster SJ, Davis JC and Hipkin LJ (1987) Insulin resistance in patients with colorectal cancer. *Br J Surg* **74**: 1031–1035

Daniell HW (1988) The influence of obesity and age at diagnosis on the estrogen receptor status of breast cancers. Perimenopausal predominance of estrogen receptor negative tumors. *Cancer* **61**: 1237–1240

Darendeliler F, Hindmarsh PC and Preece MA (1990) Growth hormone increases rate of pubertal maturation. *Acta Endocrinol* **122**: 414–416

Drafta D, Priscu A, Neacsu E, Gangura M, Schindler AE and Stroe E (1983) Estradiol and progesterone receptor levels in human breast cancer in relation to cytosol and plasma estrogen level. *J Steroid Biochem* **18**: 459–463

Ellis MJC, Singer C, Hornby A, Rasmussen A and Cullen KJ (1994) Insulin-like growth factor mediated stromal–epithelial interactions in human breast cancer. *Breast Cancer Res Treat* **31**: 249–261

Favoni RE, de Cupis A, Perrotta A, Sforzini S, Amoroso D, Pensa F and Miglietta L (1995) IGF1 and IGFBP blood serum levels in women with early and late stage breast cancer. *J Cancer Res Clin Oncol* **121**: 674–682

Feldman M, Ruan W, Cunningham BC, Wells JA and Kleinberg DL (1993) Evidence that the growth hormone receptor mediates differentiation and development of the mammary gland. *Endocrinology* **133**: 1602–1608

Foekens JA, Portengen H, Janssen M and Klijn JG (1989) IGF1 receptors and IGF1-like activity in human primary breast cancer. *Cancer* **63**: 2139–2147

Frost VJ, Helle SJ, Lonning PE, van der Stappen JWJ and Holly JMP (1996) Effect of treatment on IGF, IGFBPs and IGFBP3 protease status in patients with advanced breast cancer. *J Clin Endocrinol Metab* **81**: 2216–2221

Giovanucci E (1995) Insulin and colon cancer. *Cancer Causes Control* **6**: 164–179

Giudice LC (1995) Editorial; IGFBP3 protease regulation: How sweet it is. *J Clin Endocrinol Metab* **80**: 2279–2281

Heslin MJ, Newman E, Wolf RF, Pisters PWT and Brennan MF (1992) Effect of systemic hyperinsulinemia in cancer patients. *Cancer Res* **52**: 3845–3850

Holly JMP (1991) The physiological role of IGFBP1. *Acta Endocrinol* **124** (suppl. 2): 55–62

Jacquemier JD, Hassoun J, Torrente M and Martin PM (1990) Distribution of estrogen and progesterone receptors in healthy tissue adjacent to breast lesions at various stages; immunohistochemical study of 107 cases. *Breast Cancer Res Treat* **15**: 109–117

Juul A, Main K and Blum WF (1994) The ratio between serum levels of IGF1 and IGFBP 1, 2, 3 decreases with age in healthy adults and is increased in acromegalic patients. *J Clin Endocrinol Metab* **41**: 85–93

Khan SA, Rogers MAM, Obando JA and Tamsen A (1994) Estrogen receptor expression of benign breast epithelium and its association with breast cancer. *Cancer Res* **54**: 993–997

Kleinberg DL, Ruan W, Catanese V, Newman CB and Feldman M (1990) Non-lactogenic effects of growth hormone on growth and IGF1 mRNA of rat mammary gland. *Endocrinology* **126**: 3274–3276

McGuire SE, Hilsenbeck SG, Figueroa JA, Jackson JG and Yee D (1994) Detection of IGFBPs by ligand blotting in breast cancer tissues. *Cancer Letters* **77**: 25–32

Markopoulos C, Berger U, Wilson P, Gazet JC and Coombes PC (1988) Oestrogen receptor content of normal breast cells and breast carcinomas throughout the menstrual cycle. *Br Med J* **296**: 1349–1351

Matsumoto, K, Sakamoto G and Nomura Y (1986) International comparisons concerning breast cancer and steroid receptors. *Anticancer Res* **6**: 621–624

Mehta RR, Hart G and Das Gupta TK (1992) Steroid receptors in breast cancer patients; influence of obesity and age at diagnosis. *Anticancer Res* **12**: 1311–1314

Mol JA, Henzen Longmans SC, Hageman P, Misdorp W, Blankenstein MA and Rijnberk A (1995) Expression of the gene encoding growth hormone in the human mammary gland. *J Clin Endocrinol Metab* **80**: 3094–3096

Papa V, Gliozzo B, Clark GM, McGuire WL, Moore D, Yamaguchi FY, Vigneri R, Goldfine JD and Pezzino V (1993) IGF1 receptors are overexpressed and predict a low risk in human breast cancer. *Cancer Res* **53**: 3736–3740

Pekonen F, Partanen S, Makinen T and Rutanen EM (1988) Receptors for EGF and IGF1 and their relation to steroid receptors in human breast cancer. *Cancer Res* **48**: 1343–1347

Peyrat JP, Bonneterre J, Beuscart R, Djiane J and Demaille A (1988) IGF1 receptors in human breast cancer and their relation to estradiol and progesterone receptors. *Cancer Res* **48**: 6429–6433

Peyrat JP, Bonneterre J, Hecquet B, Vennin P, Louchez MM, Fournier C and Demaille A (1993) Plasma IGF1 concentration in human breast cancer. *Eur J Cancer* **29A**: 492–497

Railo MJ, von Smitten K and Pekonen F (1994) The prognostic value of IGF1 in breast cancer patients. Results of a follow-up study in 126 patients. *Eur J Cancer* **30A**: 307–311

Reaven GM (1988) Role of insulin resistance in human disease. Banting Lecture 1988. *Diabetes* **37**: 1595–1607

Ricketts D, Turnbull L, Ryall G, Bakshi R, Rawson NSB, Gazet JC, Nolan C and Coombes RC (1991) Estrogen and progesterone receptors in the normal female breast. *Cancer Res* **51**: 1817–1822

Rutanen EM, Stenman S, Blum W, Karkkainen T, Lehtovirta P and Stenman WH (1993) Relationship between carbohydrate metabolism and serum IGF system in postmenopausal women; comparison of endometrial cancer patients with healthy controls. *J Clin Endocrinol Metab* **77**: 199–204

Singer C, Rasmussen A, Smith HS, Lippman ME, Lynch HT and Cullen KJ (1995) Malignant breast epithelium selects for IGF 11 expression in breast stroma; evidence for paracrine function. *Cancer Res* **55**: 2448–2254

Tekden M, Kahraman H, Yucel J, Adam B, Yildiz C and Tanyeri F (1996) Insulin and C-peptide response to oral glucose in patients with breast cancer. *Ann Oncol* **7** (suppl. 5): 24

Tennant MK, Thrasher JB, Twomey PA, Birnbaum RS and Plymate SR (1996) IGFBP2 and IGFBP3 expression in benign human prostate epithelium, prostate intraepithelial neoplasia and adenocarcinoma of the prostate. *J Clin Endocrin Metab* **81**: 411–420

Vishnevsky AS, Bobrov JF, Tsyrlina EV and Dilman VM (1993) Hyperinsulinemia as a factor modifying sensitivity of endometrial cancer to hormonal influences. *Eur J Gyn Oncol* **14**: 127–130

Westley BR and May FE (1994) Role of IGF in steroid-modulated proliferation. *J Ster Biochem Mol Biol* **51**: 1–9

Yam D, Ben-Hur H, Fink A, Dgani R, Shani A, Eliraz A, Insler V and Berry EM (1994) Insulin and glucose status, tissue and plasma lipids in patients with tumours of the ovary or endometrium; possible dietary implications. *Br J Cancer* **70**: 1186–1187

Yee D (1994) The IGF system as a target in breast cancer. *Breast Cancer Res Treat* **32**: 85–95

Yoshikawa T, Noguchi Y and Matsumoto A (1994) Effects of tumour removal and body weight loss on insulin resistance in patients with cancer. *Surgery* **116**: 62–66

Yu H, Levesque MA, Khosravi MJ, Diamandi AP, Clark CM and Diamandis EP (1996) Association between IGFs and their binding proteins and other prognostic indicators in breast cancer. *Br J Cancer* **74**: 1242–1247

*© Cancer Research Campaign 1997*